# Making the FDA Great Again Requires Rebuilding America's Scientific Infrastructure

**DOI:** 10.7759/cureus.113039

**Published:** 2026-07-20

**Authors:** Arya Babul, Parisa Mahdavi

**Affiliations:** 1 Biomedical Sciences, West Career and Technical Academy, Las Vegas, USA; 2 Biomedical Sciences, Society for Awareness of Neglected Diseases, Las Vegas, USA; 3 Drug Regulatory Affairs, Tavolar LLC, Las Vegas, USA

**Keywords:** cdc surveillance, china drug development, fda regulatory policy, global health infrastructure, institutional memory, nih funding, pharmaceutical innovation, usaid, who

## Abstract

In a recent *JAMA Health Forum* commentary, Former FDA Commissioner Scott Gottlieb warns that politicizing the US Food and Drug Administration's (FDA) medical product centers risks eroding the agency's scientific credibility and surrendering its role as the global regulator of reference. He warns that turning regulatory decisions into political tools could cause the United States to lose its long‑standing role as the world's standard‑setting authority, as, at the very moment, China is positioning itself to assume that influence. While his diagnosis is correct, it does not capture the full scope of the challenge. The FDA's fragility is not an isolated political phenomenon; it reflects a broader, self‑inflicted weakening of US scientific and regulatory capacity across the National Institutes of Health (NIH), the Centers for Disease Control and Prevention (CDC), the United States Agency for International Development (USAID), and global health partnerships. At the same time, China's rapid ascent in pharmaceutical innovation is structural, not merely geopolitical, and US institutional erosion accelerates this shift. Drawing on recent analyses of China's innovation ecosystem and the importance of institutional memory in public health systems, this editorial argues that restoring FDA leadership requires rebuilding the entire scientific infrastructure that supports it. We propose a whole‑of‑system strategy centered on predictable, inflation‑adjusted funding, protection of workforce memory, and renewed engagement with global regulatory and public health networks.

## Editorial

Introduction

The mid-2026 landscape of US federal health leadership has laid bare the deep structural fragility of our health regulatory institutions [1]. In his June 2026 *JAMA Health Forum* commentary, Scott Gottlieb offers a clear warning: the politicization of FDA medical product center leadership threatens the agency's scientific integrity and global standing [2]. He writes that "politicizing the centers risks surrendering the US position as the global regulator of reference just as China builds toward replacing the FDA's international role." This concern is well‑founded.

The appointment and subsequent abrupt departure of FDA Commissioner Marty Makary occurred amid broader upheaval at the Department of Health and Human Services (HHS), including the removal of CDC Director Susan Monarez, the resignation of multiple senior CDC scientific leaders, and the dismissal of all 17 members of the CDC's Advisory Committee on Immunization Practices; together, these events highlight a profound systemic crisis within the department [3]. These cascading departures underscored how attempts to exert direct political control over career‑led review pathways can destabilize core regulatory functions rather than improve accountability. A shorter version of the argument developed in this editorial was previously discussed in a *JAMA Health Forum* response to Gottlieb's commentary, emphasizing that FDA fragility cannot be understood in isolation [2].

The FDA cannot function in a vacuum. Its historical authority and credibility have long rested on a highly specialized infrastructure of experienced career scientists who apply their technical expertise and regulatory standards consistently across administrations. However, the FDA's current challenges reflect a broader pattern of institutional erosion across the US scientific ecosystem, including upstream funding instability at the National Institutes of Health (NIH), severe workforce erosion at the CDC, the near‑total dismantling of the United States Agency for International Development's (USAID) global networks, and the United States' withdrawal from the World Health Organization (WHO). These institutions provide the evidence, surveillance, and global partnerships that underpin FDA decision‑making. Rebuilding the FDA requires a comprehensive restoration of the broader American biomedical ecosystem. To understand the FDA's current vulnerability, we must examine the degradation of the interconnected agencies that supply its evidence-based surveillance data and international legitimacy.

At the same time, China's rise in pharmaceutical innovation is not merely geopolitical positioning but a structural transformation in global drug discovery, development, and regulation. As recent analyses show, "China's ascent reflects regulatory reform, AI-enabled discovery, and the out-licensing of high-value clinical assets that increasingly shape multinational R&D pipelines" [4]. US institutional erosion accelerates this shift and undermines the country's ability to sustain its position as the world's regulator of reference.

Finally, the erosion of institutional memory across the FDA, NIH, USAID, and global health non-governmental organizations (NGOs) represents a deeper vulnerability that Gottlieb does not address. As recent work emphasizes, "when experienced field professionals and local stakeholders depart, programs lose more than people; they lose operational bandwidth, practical records, know-how, and informal problem-solving practices" [5]. Regulatory agencies do not function solely through formal rules; they rely on tacit knowledge accumulated over decades.

This editorial argues that making the FDA "great again" requires rebuilding the entire US scientific infrastructure that supports it. FDA reform must be part of a whole‑of‑system strategy to restore scientific capacity, protect institutional memory, and re-engage global partnerships.

The FDA cannot be rebuilt in isolation

These interconnected failures form the backdrop for understanding why the FDA's challenges cannot be addressed through leadership changes alone. Gottlieb correctly identifies the danger of politicizing FDA center leadership, but the FDA's ability to function as a scientific institution depends on the strength of the broader ecosystem that surrounds it. Domestic erosion across this ecosystem is the primary driver of the FDA's current vulnerability, and its effects are visible across every stage of the biomedical pipeline.

NIH: The foundation of early-stage discovery

The evidentiary base for many drug approvals begins with NIH-funded research. However, severe funding instability has introduced an unprecedented bottleneck into the drug development pipeline, disrupting clinical trials and weakening research continuity.

A landmark 2025 analysis by Patel et al. in *JAMA Internal Medicine* revealed that abrupt grant terminations affected "roughly 1 in 30 NIH-funded clinical trials, involving more than 74,000 participants," with disproportionate losses in infectious disease and prevention studies [6]. These disruptions reduce the evidence base available to FDA reviewers and weaken the pipeline of innovative therapies.

Recent federal budget proposals underscore the fragility of this foundation. The White House's FY27 budget proposal accelerates this decline, slashing NIH funding by over 10%, a drop from $47.22 billion to $41.2 billion, with the National Cancer Institute receiving an increase that does not keep pace with biomedical inflation [7-9]. Research advocacy organizations warn that such cuts endanger the speed of lifesaving cancer research. Forcing multi-year awards to be funded entirely up front has drastically reduced the absolute number of new grants awarded. These structural funding constraints weaken early‑stage discovery, destabilize research continuity, and directly erode the evidentiary base on which FDA regulatory decisions depend, particularly for complex biologics and novel platform technologies.

As a result, US‑based investigators face increasing difficulty sustaining long‑term translational programs, while multinational companies shift more early‑stage work to jurisdictions with more predictable public funding.

CDC: The backbone of surveillance

CDC workforce losses have weakened national surveillance systems that the FDA relies on to evaluate safety signals, monitor adverse events, and assess population‑level risks. Without robust surveillance, the FDA cannot effectively regulate medical products or respond to emerging threats. The FDA depends on CDC data streams for several core regulatory functions, including post‑market safety monitoring for vaccines and therapeutics, foodborne illness investigations, early detection of adverse event clusters, and situational awareness during outbreaks. CDC surveillance also informs FDA decisions related to vaccine strain selection, diagnostic test evaluation, and emergency authorizations, and supports timely recalls, label changes, and risk assessments. The scale of CDC's erosion has been documented in detail. Recent analyses describe a profound structural weakening of the agency, including a 50% proposed budget cut, elimination of major chronic disease programs, dismissal of senior scientific leaders, and the departure of more than 3,000 staff, many of them veteran field epidemiologists, virologists, laboratory scientists, and communications experts [3,8]. These losses have degraded surveillance capacity, fragmented national guidance, and undermined public trust. Such actions weaken core capacities that cannot be easily recreated, leaving the nation less prepared for outbreaks and diminishing the quality, timeliness, and completeness of the data that the FDA relies on for regulatory oversight.

USAID and WHO: Global partnerships that support regulatory credibility

Historically, the FDA's global authority was reinforced by international clinical trial networks and regulatory harmonization pathways supported in part by USAID and the WHO. Sweeping federal budget cuts have shuttered dozens of vital international field sites and brought hundreds of overseas clinical trials to an abrupt halt across Africa, Asia, and Latin America. Independent analyses show that the US withdrawal from WHO and the shutdown of USAID have disrupted global health governance, weakened disease surveillance systems, and reversed progress in major infectious disease programs [10,11]. The collapse of these global networks reduces the volume and reliability of foreign clinical evidence available to FDA reviewers, further weakening the agency's ability to assess multinational trials.

The consequences of USAID's dismantling extend far beyond program closures. Recent studies report that defunding USAID programs is projected to increase preventable mortality through 2030 and undermine clinical research capacity across low‑ and middle‑income countries [12,13]. Auwal et al. and Shaikh et al. warn that USAID's withdrawal threatens emergency preparedness, disease‑prevention programs, and the stability of global health systems [10,11]. Because the FDA participates in WHO‑coordinated regulatory harmonization forums, outbreak intelligence networks, and global pharmacovigilance collaborations, the US withdrawal from the WHO indirectly constrains the international scientific environment in which the FDA operates. WHO's emergency preparedness systems, global surveillance platforms, and regulatory strengthening initiatives have historically complemented the FDA's ability to contextualize foreign clinical evidence, monitor global manufacturing risks, and align with international norms. The loss of US engagement in these structures - combined with USAID's dismantling of field sites and trial networks - reduces the breadth and reliability of global data that once supported the FDA's evaluation of multinational trials.

A recent Lancet analysis estimated that USAID‑supported programs prevented more than 91 million deaths over two decades and that current funding cuts could result in over 14 million additional deaths by 2030 [13]. Although such mortality projections fall outside the FDA's mandate, they illustrate the scale of global health infrastructure being lost and the corresponding erosion of clinical data, trial networks, and regulatory cooperation that historically strengthened the FDA's ability to evaluate foreign evidence.

However, several critiques note that the Lancet model rests on assumptions that may not hold: it presumes future funding patterns will mirror past dynamics, underestimates system‑level shocks from abrupt donor withdrawal, and excludes concurrent cutbacks by other major donors and key programs such as polio eradication [14,15]. Even with these limitations, independent evaluations consistently show that USAID investments have been central to reducing preventable mortality, sustaining surveillance systems, and maintaining research capacity across low‑ and middle‑income countries. The current contraction of global aid - regardless of the exact mortality estimate - signals a substantial loss of international public‑health infrastructure on which the FDA has long depended for regulatory alignment and high‑quality external evidence.

Although mortality projections fall outside the FDA's direct mandate, the broad consensus among global health analysts underscores the scale of international capacity being lost and the corresponding erosion of clinical data, trial networks, and regulatory cooperation that once strengthened the FDA's ability to evaluate foreign evidence.

FDA cannot remain "great" if the institutions that supply its evidence and global legitimacy continue to erode.

China's structural rise and US vulnerability

Gottlieb warns that politicizing FDA centers risks ceding global regulatory leadership to China. However, China's rise in pharmaceutical innovation is not contingent on US missteps; it is structural. Recent analyses show that China now contributes nearly one‑third of global clinical‑stage assets, leads the world in elite scientific publications, and has rapidly expanded its portfolio of first‑in‑class and first‑approved drugs [4]. Recent approvals and new drug application (NDA) acceptances for domestically developed targeted therapies, including multiple selective JAK1 inhibitors for inflammatory skin disease, exemplify how China's regulatory and industrial ecosystem now routinely advances assets with global commercial potential. China's AI‑enabled drug discovery ecosystem has become a preferred partner for Western pharmaceutical companies seeking computationally sophisticated R&D platforms.

These trends reflect a mature innovation ecosystem characterized by coordinated industrial policy, regulatory reform, and AI‑enabled discovery platforms rather than a transient geopolitical maneuver. China's clinical research capacity has expanded at a pace unmatched by any other jurisdiction: the number of pharmaceutical clinical trial institutions nearly doubled between 2020 and 2025, and annual drug trials registered with its Center for Drug Evaluation increased from 2,386 in 2019 to 4,900 in 2024, accompanied by a rise in class 1 drug approvals from 18 to 48 over the same period [16]. Parallel analyses of biopharma R&D intensity show that multiple Chinese firms, including Hengrui, CSPC, Innovent, and Henlius, have transitioned from generic manufacturers to "innovation leaders," investing 20-27% of revenues in R&D and building pipelines of best‑in‑class and first‑in‑class assets across oncology, cardiometabolic disease, and respiratory medicine [17].

China's ascent is also driven by a deliberate startup ecosystem. China now accounts for nearly 10% of compounds in US Phase 3 trials, overtaking Japan as Asia's leading hub for drug discovery. Countries showing the strongest growth in Phase 3 share are those where post‑2000 biopharma startups contribute the majority of late‑stage candidates, a pattern most pronounced in China, where more than 80% of Phase 3 originators are startups founded after 2000 [18]. These firms disproportionately generate assets using next‑generation modalities, including bispecific antibodies, antibody‑drug conjugates, nucleic acid medicines, and cell and gene therapies.

Regulatory reform has further accelerated this trajectory. China's 2025 Order No. 818 established a dual‑track regulatory framework allowing accredited hospitals to develop and apply novel biomedical technologies through a hospital‑based pathway. By replacing formal clinical‑trial approval with record filing and authorizing pre‑market "clinical translational application" under National Health Commission oversight, China has created a promissory regulatory track that compresses translation timelines for gene and cell therapies while maintaining centralized safety oversight [19].

In oncology, China has coupled rapid innovation with aggressive access and pricing tools. Between 2015 and 2024, the National Medical Products Administration approved 158 cancer medicines, including 72 domestically branded agents, with biologics comprising more than 60% of new approvals. National Reimbursement Drug List negotiations routinely secure average price reductions exceeding 60% for high‑cost innovative drugs, while volume‑based procurement for generics has saved tens of billions of RMB for public insurance funds [20]. These mechanisms expand domestic access while strengthening commercial viability for Chinese innovators.

Industry behavior in the United States reflects growing anxiety about this shift. US and European startups increasingly withhold targets, mechanisms, and even gene names from public presentations and investors, describing China as an "existential threat" because of its ability to rapidly reverse‑engineer and optimize experimental therapies [21]. At the same time, congressional scrutiny of US‑sponsored trials in China, including investigations into studies conducted in Xinjiang and at military‑affiliated hospitals, has introduced new geopolitical friction and compliance costs that smaller biotechs describe as a "huge distraction and a huge expense" [22].

Recent commentary from former FDA leadership argues that the world is already operating in a "two‑hub" system centered on the United States and China, with first‑in‑human trials increasingly launching in China and raising difficult questions about how US regulators will handle pivotal data generated primarily outside domestic networks, especially for rare diseases [23]. These developments underscore that China's rise is structural, multifaceted, and accelerating, and that ongoing US institutional erosion further amplifies its impact.

Institutional memory: The missing dimension in FDA reform

While Gottlieb's commentary focuses heavily on appointing effective leadership, it overlooks the invisible erosion of institutional memory. Complex regulatory bodies do not operate solely on codified rulebooks; they rely on decades of accumulated tacit knowledge.

As we documented in our previous Cureus publication on rural medicine and institutional capacity, when experienced field professionals and local stakeholders depart, programs lose far more than headcount; they lose operational bandwidth, practical records, everyday know-how, and informal problem-solving practices [5].

The mass departures of career scientists across the FDA, NIH, CDC, USAID, and global NGOs represent a catastrophic loss of this "remembered craft." Appointing a strong commissioner cannot instantly replace the decades of unwritten technical expertise required to safely evaluate complex biologics, adaptive trial designs, or novel AI‑driven therapeutics. Rebuilding FDA leadership without protecting institutional memory will produce fragile reforms that cannot withstand future disruptions.

A whole-of-system strategy to restore US scientific leadership

Restoring the FDA's role as the global regulator of reference requires a whole‑of‑system strategy that strengthens the scientific institutions surrounding it. The FDA does not operate in isolation; its regulatory judgments depend on the stability and quality of upstream scientific inputs. Rebuilding US biomedical leadership, therefore, begins with restoring predictable, sustained NIH funding to support early‑stage discovery, translational research, and clinical trials. Without a strong NIH, the FDA's evidentiary foundation erodes. Equally essential is revitalizing the CDC's surveillance capacity, which provides the real‑world safety signals, epidemiologic data, and population‑level insights that inform FDA decision‑making. A regulator cannot be strong if its national surveillance backbone is weak.

Reinvestment must also extend beyond domestic agencies. USAID field sites, global health programs, and partnerships with the WHO have historically supplied the FDA with trusted international data, regulatory harmonization pathways, and collaborative networks that validate foreign clinical evidence. Rebuilding these partnerships is critical for maintaining US credibility in global regulatory science. Finally, protecting institutional memory across all these agencies is indispensable. Workforce departures at FDA, NIH, CDC, and USAID have not only reduced headcount but have also depleted tacit knowledge, including the operational know‑how, informal problem‑solving practices, and accumulated experience that make regulatory systems resilient [5]. Strengthening FDA center leadership, as Gottlieb recommends, is necessary, but it will be insufficient unless accompanied by broader investments in scientific capacity, surveillance infrastructure, global engagement, and the preservation of institutional memory.

Only a coordinated, whole‑of‑system approach can restore the scientific and regulatory foundations that once made US biomedical leadership possible. Key elements include restoring predictable, inflation-adjusted, long‑term funding; instituting comprehensive debriefing protocols and operational problem‑solving logs from departing senior scientists; replacing outdated regulatory structures that inflate costs and elongate clinical trial timelines with streamlined, rapid‑translation pathways and increased federal proof‑of‑concept funding; and immediate re‑funding of closed USAID international field sites alongside formal re‑engagement with WHO harmonization networks.

Gottlieb's call to protect FDA center leadership is, therefore, necessary but incomplete; without parallel investments in upstream science, surveillance, global engagement, and institutional memory, even the most capable commissioner will be constrained by a hollowed‑out system.

Conclusions

Scott Gottlieb's warning is an urgent one, but isolated leadership changes at the FDA will fail if the surrounding scientific infrastructure continues to hollow out. The recent crisis surrounding FDA Commissioner Marty Makary's brief tenure, occurring alongside broader leadership upheaval across HHS, demonstrates that political maneuvering at the top cannot mask a systemic collapse of the nation's scientific ecosystem. True regulatory modernization requires coordinated reinvestment in upstream basic research, robust national surveillance, global health partnerships, and the rigorous preservation of workforce memory.

At the same time, the global innovation landscape has shifted toward a two‑hub world, with early‑stage biopharmaceutical development now concentrated in the United States and China. Recent analyses show that China's share of global early‑stage drug development has risen from 8% to more than 32% in a decade, while the US share has declined from 48% to 37%. This redistribution reflects structural advantages in China's clinical research capacity, regulatory reforms, and industrial policy, not merely geopolitical competition. Absent reinvestment in the scientific institutions that support FDA decision‑making, recent US actions may inadvertently accelerate this shift, pushing multinational companies toward China's faster, more predictable development ecosystem and eroding the evidentiary base that once anchored US regulatory leadership.

Only by rebuilding the whole system can the US protect its scientific integrity, maintain its standing as a global leader in medicine, and credibly claim the FDA as a regulator of reference in an innovation landscape that is no longer merely multipolar but increasingly defined by a US-China dual‑hub structure.
